# *Eef1a2* Promotes Cell Growth, Inhibits Apoptosis and Activates JAK/STAT and AKT Signaling in Mouse Plasmacytomas

**DOI:** 10.1371/journal.pone.0010755

**Published:** 2010-05-21

**Authors:** Zhaoyang Li, Chen-Feng Qi, Dong-Mi Shin, Adriana Zingone, Helen J. Newbery, Alexander L. Kovalchuk, Catherine M. Abbott, Herbert C. Morse

**Affiliations:** 1 Laboratory of Immunopathology, National Institute of Allergy and Infectious Diseases, National Institutes of Health, Rockville, Maryland, United States of America; 2 Genetics Branch, Center for Cancer Research, National Cancer Institute, Naval Hospital, Bethesda, Maryland, United States of America; 3 Medical Genetics Section, Molecular Medicine Centre, University of Edinburgh, Western General Hospital, Edinburgh, United Kingdom; Mizoram University, India

## Abstract

**Background:**

The canonical function of EEF1A2, normally expressed only in muscle, brain, and heart, is in translational elongation, but recent studies suggest a non-canonical function as a proto-oncogene that is overexpressed in a variety of solid tumors including breast and ovary. Transcriptional profiling of a spectrum of primary mouse B cell lineage neoplasms showed that transcripts encoding EEF1A2 were uniquely overexpressed in plasmacytomas (PCT), tumors of mature plasma cells. Cases of human multiple myeloma expressed significantly higher levels of *EEF1A2* transcripts than normal bone marrow plasma cells. High-level expression was also a feature of a subset of cell lines developed from mouse PCT and from the human MM.

**Methodology/Principal Findings:**

Heightened expression of EEF1A2 was not associated with increased copy number or coding sequence mutations. shRNA-mediated knockdown of *Eef1a2* transcripts and protein was associated with growth inhibition due to delayed G1-S progression, and effects on apoptosis that were seen only under serum-starved conditions. Transcriptional profiles and western blot analyses of knockdown cells revealed impaired JAK/STAT and PI3K/AKT signaling suggesting their contributions to EEF1A2-mediated effects on PCT induction or progression.

**Conclusions/Significance:**

EEF1A2 may play contribute to the induction or progression of some PCT and a small percentage of MM. *Eef1a2* could also prove to be a useful new marker for a subset of MM and, ultimately, a possible target for therapy.

## Introduction

Cancer is a genetic disease in which tumor cells acquire the ability to proliferate uncontrollably, resist apoptosis, evade immune surveillance, and, for solid tumors, promote angiogenesis. Much of our understanding of tumor initiation and progression has resulted from the identification of genes controlling cell proliferation and apoptosis that, when aberrantly expressed, result in abnormal cell growth and malignant transformation. Considerable attention has been focused on a number of oncogenic signaling pathways that converge on a set of nuclear transcription factors. These factors, in turn, govern the activation of gene expression programs that ultimately result in malignancy. Recently, however, a number of studies have indicated that dysfunctional protein translation may also contribute to tumor development. This is perhaps best exemplified by the roles identified for the protein elongation factor, EEF1A2, in a number of human cancers [1], [2], [3], [4], [5], [6], [7].

EEF1A1 and EEF1A2 are variants of the protein elongation factor EEF1A with EEF1A1 being expressed ubiquitously while EEF1A2 is normally expressed only in heart, muscle and brain [8], [9], [10]. The canonical role for these proteins involves regulation of ribosomal polypeptide elongation by binding of amino-acylated tRNA for transport to the ribosomes [11]. EEF1A2 has also been found to have a number of non-canonical functions including phosphatidylinositol signaling [12], apoptosis [13], [14], cytoskeletal modifications [15], [16], [17], targeting proteins for degradation, and participation in the heat shock response [18], [19]. It has also been shown that EEF1A2 can transform cells and give rise to tumors in nude mice [20]. Notably, EEF1A2 has anti-apoptotic functions in certain systems, whereas EEF1A1 is a pro-apoptotic protein [14], [21].

Our interest in EEF1A2 was kindled by results from gene expression profiling of primary mouse B cell lineage tumors that revealed uniquely high expression in plasmacytomas (PCT), neoplasms of mature plasma cells [22], [23], [24], [25]. Our curiosity was heighted by the findings that EEF1A2 was also expressed at high levels in some cases of multiple myeloma (MM), a plasma cell neoplasm of humans, but not by normal plasma cells or B cells in either species. Using in vitro model systems, we found that EEF1A2 is involved in regulating cell cycle progression and survival of PCT. These data indicate that EEF1A2 may play contribute to the induction or progression of plasma cell neoplasms in both mice and humans.

## Materials and methods

### Mice, lymphomas, tissue microarray, immunohistochemistry, and oligo microarray analyses of gene expression

The origins and characteristics of primary B cell lineage lymphomas from NFS.V+ congenic, B6.λ-MYC, SJL-β2m^−/−^, IL6-TG, and BALB/c-gld/gld mice, and the techniques used for *Eef1a2* transcriptional profiling of the lymphomas using oligonucleotide arrays, were detailed previously [23], [26]. The expression of human *EEF1A2* was studied from the dataset (GSM6477 in GEO) for samples of patients with MM, monoclonal gammopathy of undetermined significance (MGUS) and normal plasma cells using Affymetrix U133A microarrays. The differences in transcript levels of *EEF1A2* between MGUS or MM and normal controls were examined by unpaired t-test with Welch's correction. A tissue microarray of costal biopsies of normal individuals or patients diagnosed with MM was purchased from Folio BioSciences (Columbus, OH). Immunohistochemical studies were detailed previously [27]. Mouse protocols were approved by Animal Care and Use Committees of the National Institute of Allergy and Infectious Diseases and the National Cancer Institute.

### Cell lines, constructs, transfection, and antibodies

The MPC11 PCT cell line was obtained from American Type Culture Collection (ATCC). MOPC315, TEPC2372, TEPC4142, PCT-AP, RPC5, ABPC4 and ABPC20 PCT cell lines were provided by Dr. M. Potter (National Cancer Institute, National Institutes of Health [NIH], Bethesda, MD). Cells were maintained at 37°C 5% CO_2_ in RPMI 1640 (Invitrogen Life Technologies) with 10% fetal bovine serum (FBS) (Hyclone), 10 mM sodium pyruvate, 1x glutamine, 1x non-essential amino acids, 0.5 µM β-mercaptoethanol and 0.1 M HEPES buffer. The human cell line, Raji, was purchased from ATCC. Cell lines ARK, MM-S1, XG-1 and XG-7 were from our laboratory and were gifts from Dr. Michael Kuehl (NCI, NIH).

*Eef1a2* cDNA was cloned from the ABPC4 cell line and was inserted into the mammalian expression vector pcDNA3.2/V5 (Invitrogen) after sequencing. The sequencing results showed no mutations. pcDNA3.2/V5-CAT (chloramphenicol acetyltransferase) and pcDNA3.2/V5 (blank vector) were used for control and mock transfection, respectively. DNA (2 µg) of each construct was used to transfect 2×10^6^ cells using transfection reagent solution V and program X-001 (Amaxa).

Specific anti-EEF1A2 polyclonal antibody was described previously [1]. Anti-EEF1A was from Upstate (Upstate Biotech, Charlottesville, VA), anti-β-actin was from Abcam, anti-V5 epitope was from Invitrogen, anti-STAT3, phosphorylated STAT3, PIK3CG, AKT and phosphorylated AKT were purchased from Cell Signaling Technology (location).

### Stable knockdown of *Eef1a2* expression

A 29mer-pRS-shRNA vector (Origene) was used to express shRNA. Sequences specific for mouse *Eef1a2* knockdown: CCTCATCTACAAGTGTGGTGGCATCGACA (shRNA-1); GTCAGCGCCTACATCA AGAAGATCGGCTA (shRNA-2); ATCTCGGGCTGGCATGGTGACAACATGCT (shRNA-3); GTGACAATGTCGGGTTCAATGTGAAGAAT (shRNA-4); control sequences: TGACCACCCTGACCTACGGCGTGCAGTGC (shRNA-C). After transfection according to the methods mentioned above, 20 µg/ml puromycin was added into medium for selection. Single cell clones were maintained in medium with 10 µg/ml puromycin.

### Western blot

Total cell lysates were prepared in RIPA lysis buffer (Pierce Chemical Co.) supplemented with protease inhibitor cocktail solution (Pierce Chemical Co.). Lysates were cleared by centrifugation at 13,000 g for 15 min at 4°C, and the protein content was determined using the BCA protein assay kit (Pierce Chemical Co.). 15 µg of protein per lane was separated on a NuPage 12% Bis-Tris gel (Invitrogen) and transferred to a polyvinylidene difluoride membrane (Invitrogen). After blocking with a 5% skim milk solution, the blot was incubated with indicated antibodies. The primary antibodies were detected with horseradish peroxidase–conjugated secondary antibody (R&D system) and developed by Super Signal West pico detection kit (Pierce Chemical Co.) according to the manufacturer's instructions.

### Cell cycle and proliferation assay

1×10^6^ cells expressing the *Eef1a2* RNAi or control cells were harvested, washed with PBS, fixed with 70% ethanol overnight at minus 20°C, and treated with 10 µg/mL RNase (Roche). Cells were then stained with propidium iodide (PI) (5 µg/mL) and the cell cycle profile was determined using a FACSCalibur flow cytometer (Becton Dickinson, Mountain View, CA). Data are representative of three independent experiments and were analyzed with the FlowJo software (Tree Star, Inc., Ashland, OR).

For cell proliferation assays, the Click-iT EdU Flow Cytometry Assay Kit (Invitrogen), which is a BrdU alternative assay kit, was used according to the manufacturer's instructions. Briefly, 1×10^6^ cells/well were cultured overnight in 24-well plates. 10 µM EdU were then added to each well. 4 hours later, the cells were harvested and permeabilized immediately. After incubation with anti-EdU for 30 min, the cells were analyzed by flow cytometry.

### Apoptosis assay

Apoptotic cells were differentiated from viable or necrotic cells by combined application of Annexin V-FITC and PI using the Vybrant Apoptosis Assay Kit #3 (Invitrogen). Briefly, cells were centrifuged and the cell pellet was suspended in 1x Annexin V binding buffer at a concentration of 1×10^6^ cells/ml. Samples were incubated with 0.5 µg/ml Annexin V-FITC and 2 µg/ml PI for 10 min at room temperature and then were examined by flow cytometry. Data are representative of three independent experiments.

### Quantitative Real-Time PCR (qPCR) and qPCR arrays

Total RNA was isolated using the RNeasy mini kit coupled with DNase set (both from Qiagen). Reverse transcription was performed using 1 µg of RNA, 300 ng random hexamer primer (Invitrogen), and 200 units Superscript II (Invitrogen). The primers for qPCR were designed using Primer Express software (Applied Biosystems, Foster City, CA) and synthesized at Integrated DNA Technologies, Inc (Coralville, IA; Table S1). Each qPCR reaction was performed in a mix of 10 µl reaction mixture containing 2 ng of cDNA, 2xSYBR Green PCR Master Mix (Applied Biosystems), and 0.3 µM of each forward and reverse primer on the ABI PRISM 7900HT sequence detector system (Applied Biosystems). All samples were tested in triplicate, and analyses were performed using SDS v2.2 software (Applied Biosystems) according to the manufacturer's instruction. The comparative CT method (ΔΔCT) was used for quantification of gene expression. A single product for each primer pair was confirmed by gel electrophoresis and melt-curve analyses. The primers used for qPCR are listed in Table S1.

For qPCR array assays, cDNAs were applied to 384-well plates containing predetermined primer pairs representing various genes known to be cancer related and/or important for lymphoid cell development and function (Bar Harbor BioTechnology, Trenton, ME). PCR amplification was performed using regular SYBR-Green reagents (Applied Biosystems) and analyzed by a global pattern recognition algorithm as recently modified (http://array.lonza.com/apps/gpr/) [28]. The differentially expressed genes were classified by GO (Gene Ontology), and enrichment of significant genes was determined by Fisher's exact test.

## Results

### Expression of *Eef1a2* in primary mouse PCT

**Figure 1:**
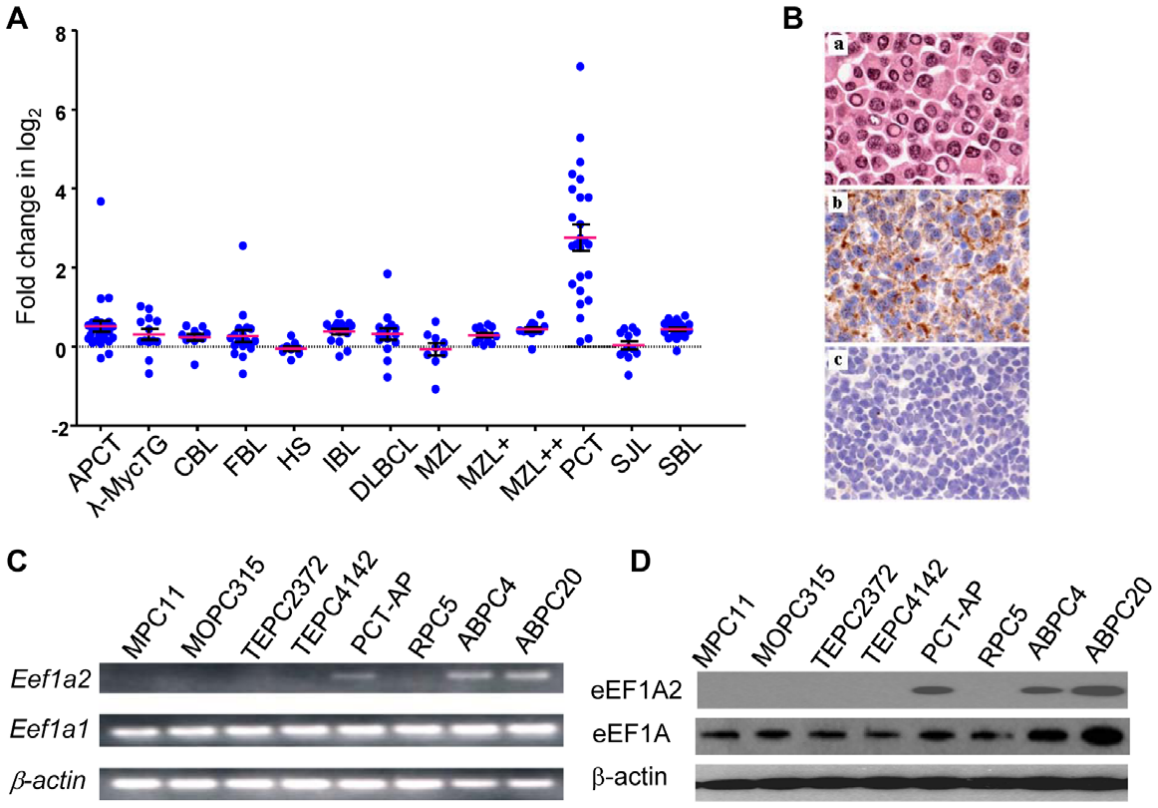
Expression of *Eef1a2* in primary mouse lymphomas and PCT cell lines. (**A**) Microarray analyses of *Eef1a2* expression were performed among 11 classes of mouse primary B cell lymphomas and histiocytic sarcomas: APCT, anaplastic plasmacytoma [22]; λ-MycTG, lymphomas from λ-Myc transgenic mice [46]; CBL, centroblastic lymphoma [47]; FBL, follicular B cell lymphoma [48]; HS, histiocytic sarcoma [47]; IBL, immunoblastic lymphoma [47]; DLBCL, diffuse large B cell lymphoma [47]; MZL, marginal zone lymphoma (low grade); MZL+ and MZL++, marginal zone lymphoma (high grade) [48]; PCT, plasmacytoma [49]; SJL, SJL mouse lymphoma [23]; SBL, small B cell lymphoma [47]. Each point represents a single tumor. The red line indicates the mean value for all tumors. The error bar  =  ± S.D. (**B**) Immunohistochemical studies of EEF1A2 expression using a polyclonal antibody specifically recognizing eEF1A2. a, H&E staining of PCT; b, IHC staining of PCT, c, IHC staining of MZL. (**C, D**) RT-PCR and Western blot analyses of *Eef1a2* expression at the transcript (C) and protein (D) levels in PCT cell lines.

Eleven classes of mouse primary B cell lineage lymphomas were analyzed using oligonucleotide microarrays that queried over 11,000 genes. A *t* test was used to identify genes that distinguished each subset from all the others. These studies identified *Eef1a2* as one of the genes highly expressed in PCT (Figure 1A), with mean transcript levels in PCT being around five-fold higher than in the other tumor classes. RT-PCR analyses of *Eef1a2* expression in primary PCT, four other lymphoma subsets and normal spleen tissue showed that *Eefla2* transcripts were detected only in samples from PCT (data not shown). Immunohistochemical studies using an EEF1A2-specific polyclonal antibody showed that EEF1A2 was expressed at high levels in the cytoplasm of PCT but not in MZL or other types of lymphomas (Figure 1B and data not shown). qPCR analyses of sorted mouse plasma cells and B220^+^ splenic B cells revealed that transcript levels for *Eef1a2* were below the levels of detection in both populations (data not shown), an observation consistent with earlier studies showing that EEF1A2 is normally expressed only in heart, muscle and brain. These studies established that among primary B cell lineage neoplasms of mice, EEF1A2 is uniquely expressed at high levels in PCT, and that expression might be linked to pathogenesis because it is not expressed by normal plasma cells.

### Expression of *Eef1a2* in PCT cell lines

Recently, gene expression profiles were generated for six subtypes of pristane-induced mouse PCT. The groups included tumors induced by pristane alone as well as those from pristane-treated mice injected with acutely transforming retroviruses [29]. High-level expression of *Eef1a2* was found in all of the subtypes (data not shown) indicating that expression of *Eef1a2* in PCT was independent of the mode of PCT induction. We next analyzed EEF1A2 expression at the transcript and protein levels in eight PCT cell lines (Figure 1C, D). RT-PCR analyses showed that three lines (ABPC4, ABPC20 and PCT-AP) had substantial levels of *Eef1a2* transcripts, while all eight expressed equivalent levels of transcripts for the closely related gene, *Eef1a1*, using *β-actin* transcript levels as a control (Figure 1C). Western blot analyses of EEF1A2 and EEF1A expression in the eight lines (Figure 1D) showed that EEF1A2 protein was present in protein extracts from the same tumors that were positive by RT-PCR. Comparative genomic hybridization (CGH) analyses of ABPC4 and ABPC20 cell lines showed no increase in copy number for the region of chromosome 2 where *Eef1a2* resides (data not shown). These data indicated that high level expression of EEF1A2 by PCT was independent of their mode of induction and was not based on increased copy number, at least in the cases examined.

### Expression of *EEF1A2* in purified plasma cells from normal bone marrow, from individuals with MGUS or primary MM cells and from MM cell lines

**Figure 2:**
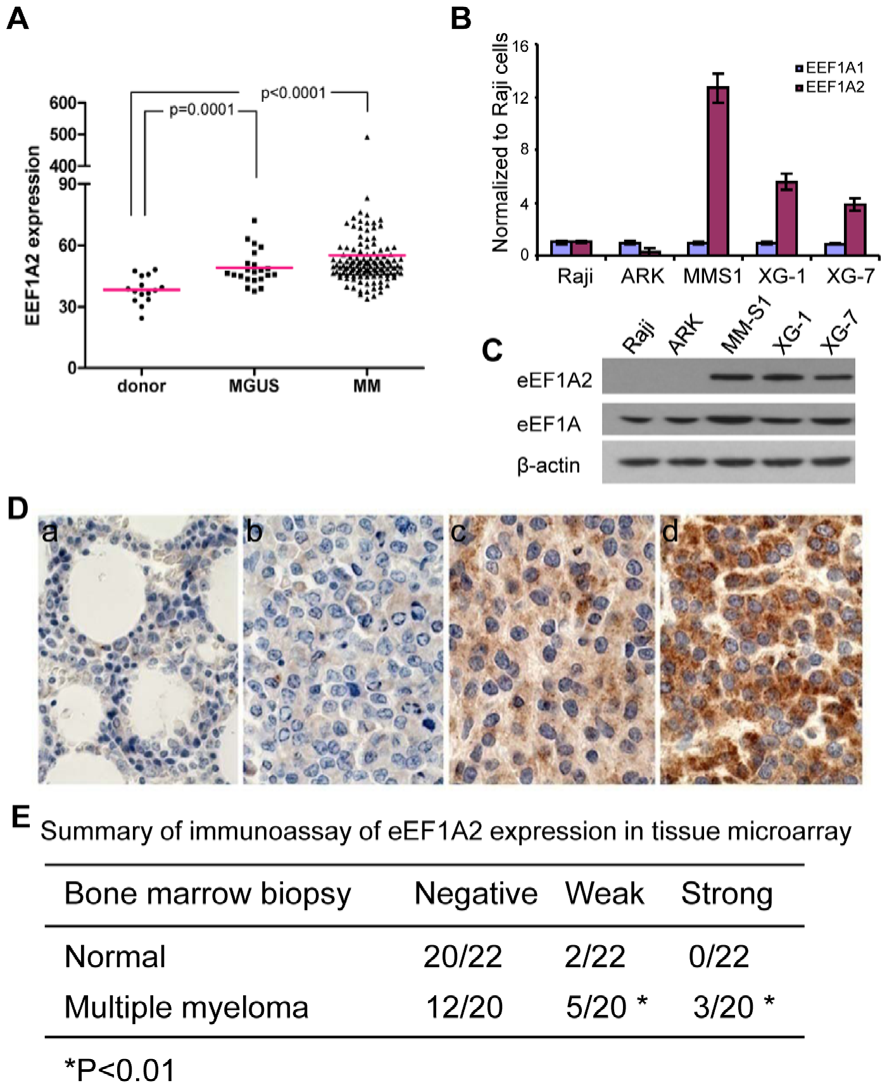
Expression of *EEF1A2* in human MM and MM cell lines. (**A**) Expression level of *EEF1A2* in human MM determined by microarray. RNA from CD138+ plasma cells from the bone marrow of patients with MGUS, MM and donor, was hybridized to Affymetrix U133A microarrays. Data was from GEO (GSM6477). Unpaired t test with Welch's correction was carried out between MGUS or MM and donors. (**B, C**) RT-PCR and Western blot analyses of *EEF1A2* expression at the transcript (B) and protein (C) levels in the indicated human MM cell lines. The error bar  =  ± S.D. (**D**) Immunohistochemistry analyses of EEF1A2 expression in normal costal tissue with negative staining (a) of EEF1A2 and MM tissues with negative staining (b), weak staining (c) and strong staining (d) of EEF1A2. (E) Summary of immunoassay of EEF1A2 expression in tissue microarray.

We next used published gene expression profiling (GSM6477 in GEO)[30] to study *EEF1A2* transcript levels in sort-purified CD138^+^ bone marrow plasma cells from 15 normal controls, 22 patients with MGUS and 125 primary cases of MM (Figure 2A). While *EEF1A2* transcript levels were very low in normal controls, the levels in plasma cells from individuals with MGUS, non-transformed precursors to probably all cases of MM, were significantly higher (p = 0.0001). Furthermore, the levels in MM were also significantly higher than normal controls (p<0.0001) but were not significantly higher than for MGUS. High levels of *EEF1A2* transcripts and protein were confirmed for three human MM cell lines by qPCR and western blot analyses, respectively (Figure 2B, 2C).

We extended these analyses of MM by immunohistochemical studies using a tissue microarray containing bone marrow biopsies from 20 normal controls and 20 patients with MM. The results showed that EEF1A2 protein was detected at background to low levels in biopsies from normal controls and five cases of MM but at high levels in 15% of primary MM (Figure 2D, 2E), This suggests that levels of EEF1A2 expression in primary MM tumor cells may be determined post-transcriptionally as well as translationally and clearly deserves further study. We conclude that expression of *EEF1A2* is progressively upregulated during the progression of normal plasma cells to MGUS and MGUS to MM.

### Transient and stable silencing of *Eef1a2* in PCT cell lines

To understand the consequences of high-level expression of EEF1A2 in PCT, we transiently transfected the PCT-AP PCT cell line with 4 different shRNAs directed at *Eef1a2* as well as a control shRNA and quantified *Eef1a2* transcript levels by qPCR. As shown in Figure S1A, the expression of *Eef1a2* in was markedly downregulated in cells transfected with shRNA-3 and shRNA-4 while the other two shRNAs were much less effective and the control, shRNA-C, had no effect. We also generated stable transfectants of shRNA-3 and the control shRNA-C in the ABPC4 PCT cell line following selection with puromycin. The *Eef1a2* knockdown cell line had barely detectable levels of *Eef1a2* transcripts (Figure S1B, left panel) and EEF1A2 protein (Figure S1B, right panel), but normal levels of *Eef1a1* transcripts and relatively normal levels of EEF1A protein (data not shown). This indicated that *Eef1a2* transcripts were specifically silenced by the targeting shRNA-3 in APBC4.

### Knockdown of *Eef1a2* inhibits cell growth and cell proliferation

**Figure 3:**
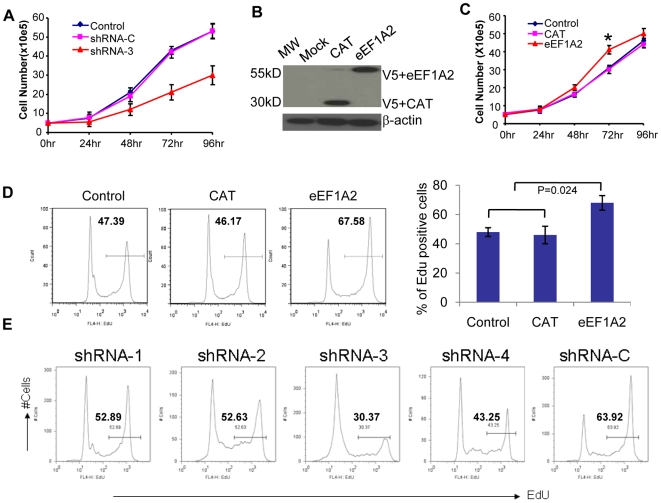
Knockdown of *Eef1a2* inhibits cell growth and cell proliferation. (**A**) ABPC4 cell numbers stably expressing *Eef1a2* shRNA-3 and control shRNA-C cells were determined during four days in culture. (**B, C**) Overexpression of EEF1A2 protein in the MPC11 cell line (B). Cell numbers were determined during culture after transfection (C). (**D**) The frequency of EdU-postive MPC11 cells in transiently transfected *Eef1a2* and control plasmid was analyzed by flow cytometry (left). The statistic bars show on the right. (**E**) The frequency of EdU-postive PCT-AP cells in transiently transfected *Eef1a2* shRNAs, and control shRNA expressing PCT-AP cells was analyzed by flow cytometry.

It was reported that human EEF1A2 promoted cell growth and proliferation in human ovarian cancer. To examine the relationship between EEF1A2 expression and PCT growth rates, we compared cell numbers of ABPC4 expressing control shRNA-C or *Eef1a2* shRNA-3 during four days in culture (Figure 3A). The growth of cells expressing shRNA-3 was significantly reduced after 72 h and 96 h, with cell numbers in these cultures being only half that of untransfected cells or cells transfected with shRNA-C.

The observed changes in cell number could be due to reduced cell cycle progression, increased apoptosis or both. To examine these possibilities, we first evaluated cells for EdU uptake after 4 h in culture using stably transfected cell lines. These studies showed that the frequency of EdU-positive cells in the *Eef1a2* shRNA-3 expressing cells was about half that of normal cells or cells expressing the inactive shRNA (Figure S2A). To extend this observation, we analyzed cell proliferation in transiently transfected cells from a second PCT cell line, PCT-AP. The results shown in Figure 3D indicated that knockdown of *Eef1a2* was associated with inhibition of PCT-AP proliferation, and that the inhibitory effect was related to the knockdown efficacy of the individual shRNAs (Figure 3E). Taken together, these results indicated that EEF1A2 promotes cell growth in the two PCT cell lines examined.

These observations prompted us to see if the growth of a PCT cell line, MPC11, that does not express EEF1A2 (Figure 1C, D), would be enhanced by introducing an EEF1A2 expression vector. To this end, we transiently expressed mouse *Eef1a2* or *CAT*, as a control, in MPC11 under the control of the CMV promoter. The *Eef1a2* and CAT constructs were tagged at the carboxy termini with the V5 epitope to facilitate detection by western blotting. Equivalent levels of EEF1A2 and CAT protein were expressed in the transfected cells (Figure 3B). The growth of EEF1A2 expressing cells was only modestly increased over those expressing CAT, best seen at 72 hr [P<0.05] (Figure 3C). Edu binding assay also showed increased proliferation (Figure 3D). Together, our results demonstrated that expression of EEF1A2 enhanced proliferation of PCT cell lines.

### *Eef1a2* knockdown delays cell cycle entry

**Figure 4:**
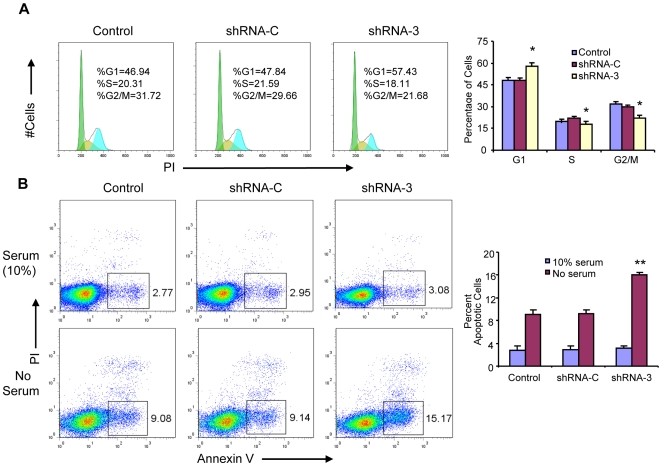
*Eef1a2* knockdown delays cell cycle entry and increases apoptosis induced by serum-free medium. (**A**) Cell cycle analyses of PI-stained *Eef1a2* shRNA and control cells using flow cytometry. (**B**) Early stage apoptotic cells were analyzed by flow cytometry in stably expressing *Eef1a2* and control shRNAs and control cells after culturing in serum-free medium for 12 hours. Error bar  =  ± S.E. *p<0.05, **p<0.01.

Next, we asked if the effect of EEF1A2 on cell growth might also be related to altered cell cycle regulation. Untransfected ABPC4 cells and cells with the active and inactive shRNAs were stained with PI and examined by flow cytometry (Figure 4A). *Eef1a2* knockdown cells had significantly increased percentages of cells in G1 and significantly decreased proportions of cells in both the S and G2/M stages of the cell cycle (Figure 4A), indicating that down regulation of *Eef1a2* delayed G1 to S progression to a limited but significant extent. Moreover, the absence of a sub-G1 accumulation showed that apoptosis was not significantly increased after knockdown of *Eef1a2* expression with cells grown in serum-containing medium.

### Serum-free conditions enhance apoptosis in *Eef1a2* knockdown cells

Pro- or anti-apoptotic effects of some genes become evident only under conditions of cellular stress. This promoted us to determine if depletion of EEF1A2 in ABPC4 might affect the viability of cells cultured in serum-free medium. We therefore examined cells stained with Annexin V and propidium iodide by flow cytometry to determine early apoptotic signaling. After 12 h in serum-free medium, 15% of *Eef1a2* knockdown cells were found to be undergoing apoptosis as compared to ∼9% of cells in both the control cell line and the control RNAi cell line (Figure 4B). Studies of the sub-G1 peak in these cultures using flow cytometry to examine cells stained with PI yielded similar results (data not shown). Similar results were found using a second transiently transfected cell line, PCT-AP (Figure S1B). These results indicated that knockdown of *Eef1a2* expression enhanced apoptosis induced by serum starvation in PCT cell lines, but only modestly.

### Knockdown of *Eef1a2* expression alters expression of many genes involved in proliferation and signaling

**Figure 5:**
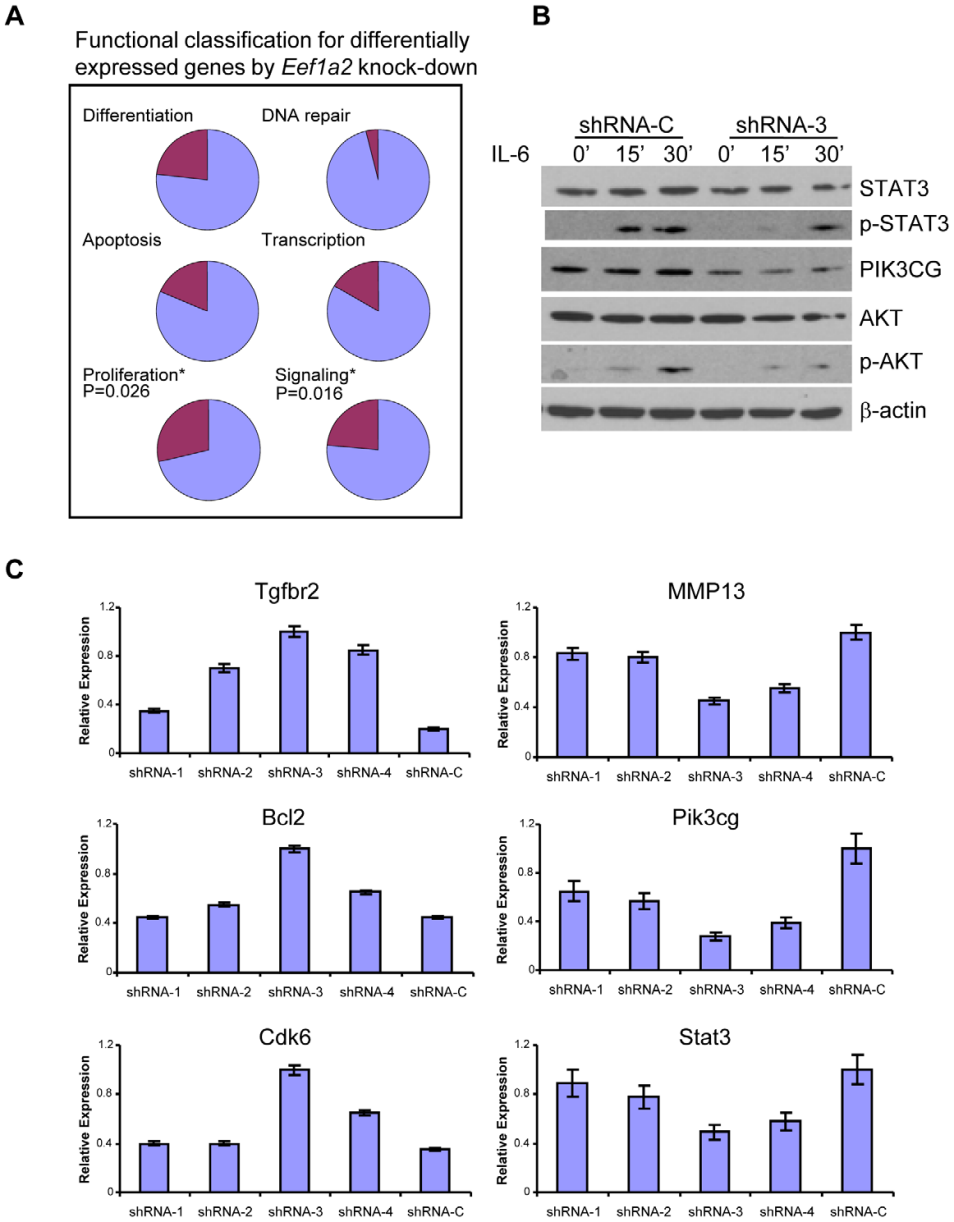
Functional changes after *Eef1a2* knockdown. (A) Functional classification of differentially expressed genes by *Eef1a2* knockdown cells. (B) Downregulation of *Eef1a2* impaired IL-6-induced AKT and STAT3 phosphorylation. *Eef1a2* shRNA and control cells were treated with 100 ng/ml recombinant IL-6 and protein samples were prepared 15 and 30 min later. Western blot analyses were performed using the indicated antibodies. (**C**) qPCR analyses of gene expression levels in transiently transfected shRNAs in PCT-AP cell line with four plasmids expressing specifically targeting *Eef1a2* (shRNA-1,2,3,4) and a control plasmid (shRNA-C). Error bar  =  ± S.E.

Apart from its canonical function, it is known that EEF1A2 can activate the AKT signaling pathway by binding directly to PI4K2B [12], [31], [32]. To gain further insight into the consequences of *Eef1a2* knockdown in PCT, we performed gene expression profiling using a 384 well qPCR array enriched for genes known to be involved in hematopoietic neoplasms. Genes for which expression was substantively altered in the stable *Eef1a2* knockdown cell line are listed in Figure 5A in relation to their functional categorization by gene ontology and analyses of enrichment within each category. Significant enrichment was observed for genes involved in proliferation (p = 0.016), in keeping with the data presented above, and signaling (p = 0.026). Among the genes involved in signaling, there was significant enrichment specifically in genes involved in the JAK/STAT signaling pathway (p = 0.010) (Table S2). These results showed that EEF1A2 might be involved in the regulation of JAK/STAT signaling as well as the AKT pathway as reported previously [12], [20], [32].

To examine these possibilities, we treated the *Eef1a2* knockdown cell line and control RNAi cell line with IL-6, and examined them for expression of STAT3, pSTAT3, AKT, pAKT and β-actin (Figure 5B). The results showed that phosphorylated STAT3 was readily detectable after 15 min in control RNAi cells, but reached similar levels only after 30 min in *Eef1a2* RNAi cells. pAKT was detectable in both cell lines at very low levels at 15 min after stimulation, but increased substantially at 30 min only in control cells. These results indicated that knockdown of *Eef1a2* expression delayed or impaired IL-6-induced activation of both the STAT3 and AKT signaling pathways. Importantly, the protein level of PIK3CG, which is upstream of AKT, was significantly decreased in the *Eef1a2* RNAi cell line at all time points. Furthermore, transcript levels of *Pik3cg* were decreased in *Eef1a2* RNAi cells (Table 1) indicating that control of PIK3CG protein expression was determined at the transcriptional level.

**Table 1:** Genes expressed at significantly increased or decreased levels in *Eef1a2*-knockdown cell line compared to control cell line.

Gene Name	Fold Change (up)	Gene Name	Fold Change (down)
*Tgfbr2*	7.47	*Mmp13*	−8.29
*Bcl2*	4.68	*Pik3cg*	−4.67
*Cdk6*	4.12	*Ifi202b*	−4.28
*Tnfrsf17*	4.13	*Lmo2*	−4.51
*Ltb*	4.98	*Cd38*	−12.78
*Sox4*	4.2	*Cd28*	−3.89
*Bcl9*	4.34	*Tnfsf13b*	−4.36
*Ptgs1*	2.88	*Fosb*	−2.55
*Smo*	4.03	*Itgal*	−2.43
*Hoxa1*	13.17	*Stat1*	−2.39
*Ikzf3*	3.97	*Rarg*	−2.57
*Ccr7*	3.5	*Dkk1*	−4.8
*Id3*	2.57	*B2m*	−1.76
*Smad7*	8.67	*Gli2*	−3.92
*Hoxa9*	4.24	*Flt3*	−1.71
*Il18*	2.52	*Hes1*	−2
*Xrcc2*	2.83	*Fos*	−1.97
*Six3*	2.94	*Dtx1*	−3.05
*Wnt2*	5.06	*Ly6a*	−1.75
*Ccnd2*	1.78	*Irf2*	−2.01
*Hes7*	2.91	*Wnt1*	−6.99
*Ccl3*	4.8	*Irf8*	−2.58
*Lyn*	1.57	*Socs3*	−2.09
*Ifnb1*	2.63	*Id2*	−1.66
*Traf1*	1.74	*Jak2*	−1.49
*Cd86*	8.59	*Stat2*	−1.9
*Bcl2l1*	1.65	*Spn*	−1.85
*IgL-C1*	1.69	*Stat3*	−1.53
*Fyn*	1.58	*Map2k1*	−1.68
*Mbd1*	1.49	*Jag2*	−4.27
*Cd79a*	1.48	*Hoxa13*	−2.05

RNA prepared from purified cells of *Eef1a2* RNAi and control RNAi was assayed by qPCR, and genes exhibiting significant changes (*p*<0.05) were identified using a modified GPR™ algorithm.

Among the genes with significantly altered expression in *Eef1a2* RNAi cells (Table 1) were those involved in tumor invasion (*Tgfbr2*, *Mmp13*, *Itgal*), proliferation (*Pik3cg*, *Fosb*, *Fos*, *Mapk1*, *Flt3*, *Wnt1*, *Cdk6*, *Ccnd2*), survival (*Ifi202b*, *Tnfsf13b*, *Bcl2*, *Bcl2l1*), and cytokine and interferon signaling (*Jak2*, *Stat1*, *Stat2*, *Stat3*, *Irf2*, *Irf8*, *Socs3*). Most interestingly, a number of the genes contain SH2 or SH3 domains - JAK2, STAT1, STAT2, STAT3, SOCS3, FYN, LYN, and PIK3CG - supporting the suggestion from previous studies that EEF1A2 may directly or indirectly interact with proteins which containing SH2 or SH3 domains [33], [34]. Furthermore, increased levels of transcripts for *Bcl2* and *Bcl2l1* may affect the limited degree of apoptosis associated with knockdown of *Eef1a2* expression.

To further validate our qPCR array data, we analyzed the expression of *Tgfbr2*, *Bcl2*, *Cdk6*, *MMP13*, *Pik3cg* and *Stat3*, genes that are significantly regulated in ABPC4 cells with a stable knockdown of *Eef1a2* (Table 1) or using PCT-AP cells transiently transfected with knockdown and control shRNAs (Figure 5C). After transfection for 48 hours, all six genes showed changes in expression that were consistent with the data obtained from stably transfected cells (Table 1). In addition, some of the regulatory effects could be related to the knockdown efficacy of the individual shRNAs (Figure S1A). We conclude that EEF1A2 affects the expression of a number of signaling pathways.

## Discussion

In tissues of normal animals, EEF1A2 is expressed only in heart, brain and muscle. The data presented here indicate that *EEF1A2* is aberrantly expressed at high levels in some plasma cell neoplasms of mice and humans. This is the first example of EEF1A2 being expressed in a mouse tumor, although high-level expression has been documented in a variety of human tumors belonging to different cell lineages [1], [2], [3], [4], [5], [6], [7]. In contrast to earlier studies suggesting that increased expression of *EEF1A2* is associated with terminal differentiation [35], normal mouse and human plasma cells were found to be EEF1A2-negative. Heightened expression cannot be tied uniquely to transformation within the B cell lineage as other subsets of mouse B cell tumors did not express *Eef1a2*. Furthermore, additional studies, although limited, suggest that heightened expression in these tumors is not due to mutation or amplification of the gene. Previous studies of ovarian tumors that aberrantly express *EEF1A2* at high levels also ruled out contributions of mutation or changes in the methylation status of the gene, and showed that levels of expression did not correlate with gene amplification [36]. Interestingly, heightened levels of *EEF1A*2 transcripts in MM related most closely to a subset of cases that lack primary IgH translocations and overexpress D-type cyclins. Features of this MM subset that might drive *EEF1A2* expression remain to be determined.

The observations that *Eef1a2* transcripts are expressed at increased levels in the majority of primary PCT but in a smaller proportion of primary MM indicate that the mechanisms governing aberrant expression and possible contributions to transformation of plasma cells are likely to differ between the species. Whether these differences are cell intrinsic or species-dependent remain to be determined.

Our studies of mouse PCT suggest that heightened expression of EEF1A2 might contribute to transformation by promoting cell cycle progression and inhibiting apoptosis. Support for this view comes from prior studies of non-PCT cell lines overexpressing EEF1A2 that exhibited enhanced cell growth [20] and resistance to apoptosis [14], [21]. Our studies of PCT cell lines suggest that activation of STAT3 and AKT may contribute to inhibition of apoptosis.

Although several mouse models have emerged as useful platforms for mechanistic and therapeutic studies of alterations in signaling pathways found in human MM (e.g., IL-6, *Abl*, and *Myc*), none of the models faithfully recapitulates all features of human MM. We screened 11 types of mouse lymphoma by microarray and found *Eef1a2* as a candidate cancer gene that was expressed at high levels only in PCT. None of the other classes of B cell lineage tumors expressed *Eef1a2* at high levels, including anaplastic and plasmablastic PCT, which have a number of histological and molecular similarities to plasmacytic PCT [22], [37].

Since *Eef1a2* is not expressed in normal plasma cells, our findings suggest that inappropriate expression of EEF1A2 in B cell differentiation may contribute to the induction, progression or survival of a high proportion of primary PCT. *Eef1a2* was expressed at high levels by PCT from mice of four different genetic backgrounds that had been subjected to a variety of PCT induction protocols. We also found *EEF1A2* was highly expressed at the protein level in 15% of primary cases of MM as well as a number of MM cell lines. Interestingly, levels of *EEF1A2* transcripts were increased to a lesser extent in plasma cells of individuals with MGUS, a consistent precursor to MM [38], than in primary MM. This suggests that activation occurred during the transition of normal plasma cells to MGUS with the levels in cases of primary MM not being significantly higher than in MGUS. These observations indicate that deregulated expression of EEF1A2 might be a common contributor to the pathogenesis of mouse PCT and a subset of human MM in ways affecting disease initiation although contributions to progression are also possible.

There is increasing evidence to indicate that EEF1A2 is a candidate oncogene, highly expressed in some human breast, ovarian, and lung cancers. It has been very difficult, however, to relate genetic features of these cancers with levels of EEF1A2 protein expression. In human solid tumors, for example, increases in copy number did not correlate with levels of protein expression. Indeed several tumors with amplification of the *EEF1A2* gene did not express the protein while other tumors without amplification expressed the protein at high levels. Possible contributions of epigenetic regulation to expression are suggested by studies showing that treatment of gastric cancer cell lines with demethylating agents resulted in increased *EEF1A2* expression [39]. We showed that overexpression of *Eef1a2* in PCT was not due to increased copy number in the few cases examined nor to mutation of the *Eef1a2* coding sequence cloned from one of the high expressing PCT. Sequencing studies of *Eef1a2* transcripts in other tumors would clearly be required to fully exclude the possibility that coding region mutations might affect protein expression. In normal tissues, expression of *Eef1a2* is restricted to muscle, brain, and heart, and neither normal plasma cells nor B cells express transcripts or protein. Developing an understanding of the basis for activation in PCT and MM is an important target for ongoing studies.

Previous studies showed that overexpression of human EEF1A2 in NIH3T3 cells enhanced cell growth [20], while overexpression in a mouse myoblast cell line protected against caspase-3-meditated apoptosis, indicative of a pro-survival function [14]. More recently, it has been shown that expression of EEF1A2 together with PRDX1 rendered NIH3T3 cells dramatically resistant to apoptosis induced by oxidative stress [21]. In PCT, we demonstrated that overexpression of *Eef1a2* enhanced cell proliferation and that *Eef1a2* knockdown cells, while not undergoing apoptosis under normal serum conditions, became more sensitive to death following serum withdrawal. Delayed cell cycle entry from G1/G0 to S in PCT following knockdown of EEF1A2 expression might be related to decreased rates of protein synthesis.

IL-6 signaling is known to be critical to the growth and differentiation of normal plasma cells as well as PCT and MM. Pathways downstream from the receptor include the JAK/STAT pathway and the PI3K-AKT-mTOR pathway. Our studies indicate that EEF1A2 may modulate both pathways since phosphorylation of STAT3 and AKT was delayed and protein levels of the regulatory subunit of PI3K were significantly reduced in the knockdown cells. The results with AKT are consistent with previous studies showing that EEF1A2 can directly activate AKT [12]. The effects on STAT3 activation with its known importance in MM are again in keeping with a possible contribution of EEF1A2 to plasma cell transformation [40], [41], [42], [43], [44], [45].

It is worth noting that many of the EEF1A2-mediated changes that we have described as possibly contributing to transformation are affected at the transcriptional level and represent non-canonical functions of the protein. The canonical activity of EEF1A2 is clearly as a translation factor, and the effects of depletion on cell cycle entry can almost certainly be ascribed to this activity. It is more than likely that understandings of possible EEF1A2 contributions to plasma cell transformation might be appreciated more readily by approaches other than transcriptional profiling.

Taken in the light of these previous reports, our results strengthen the suggestion that *Eef1a2*, as a proto-oncogene, is involved in the growth and proliferation of PCT and possibly MM through direct or indirect regulation of the JAK/STAT and AKT signaling pathways. Further mechanistic studies of this molecule should provide new insights into the pathogenesis of these plasma cell neoplasms. *EEF1A2* could also prove to be a useful new marker for a subset of MM and ultimately, be considered as a target for therapy in cases expressing high levels of protein.

## Supporting Information

Figure S1 Specific knockdown *Eef1a2* expression in PCT cell lines. (A) qPCR analyses of *Eef1a1* and *Eef1a2* expression levels in transiently transfected PCT-AP cell line with four plasmids specifically targeting *Eef1a2* (shRNA-1,2,3,4) and a control plasmid (shRNA-C). (B) *Eef1a1* and *Eef1a2* transcripts (left) and eEF1A2 protein (right) levels were analyzed by qPCR and western blotting, respectively, in stably transfected ABPC4 cells with a plasmid expressing shRNA-3 specifically targeting *Eef1a2* and a plasmid expressing control shRNA-C. Error bar  =  ± S.E. **p<0.01.
(0.02 MB PDF)

Figure S2 (A) The frequency of EdU-postive cells in the *Eef1a2* shRNA-3 expressing, control shRNA-C expressing cells and control cells were analyzed by flow cytometry. Error bar  =  ± S.E. **p<0.01. (B) Apoptotic cells were analyzed by flow cytometry in cells transiently transfected with *Eef1a2* shRNAs and shRNA-C after culturing in serum-free medium for 48 hours.
(0.05 MB PDF)

Table S1 Primers used for qPCR.
(0.08 MB PDF)

Table S2 Signaling classification for differentially expressed genes by *Eef1a2* knock-down.
(0.10 MB PDF)

## References

[1] Tomlinson VA, Newbery HJ, Wray NR, Jackson J, Larionov A (2005). Translation elongation factor eEF1A2 is a potential oncoprotein that is overexpressed in two-thirds of breast tumours.. BMC Cancer.

[2] Li R, Wang H, Bekele BN, Yin Z, Caraway NP (2006). Identification of putative oncogenes in lung adenocarcinoma by a comprehensive functional genomic approach.. Oncogene.

[3] Kulkarni G, Turbin DA, Amiri A, Jeganathan S, Andrade-Navarro MA (2007). Expression of protein elongation factor eEF1A2 predicts favorable outcome in breast cancer.. Breast Cancer Res Treat.

[4] Grassi G, Scaggiante B, Farra R, Dapas B, Agostini F (2007). The expression levels of the translational factors eEF1A 1/2 correlate with cell growth but not apoptosis in hepatocellular carcinoma cell lines with different differentiation grade.. Biochimie.

[5] Sun Y, Wong N, Guan Y, Salamanca CM, Cheng JC (2008). The eukaryotic translation elongation factor eEF1A2 induces neoplastic properties and mediates tumorigenic effects of ZNF217 in precursor cells of human ovarian carcinomas.. Int J Cancer.

[6] Kido T, Lau YF (2008). The human Y-encoded testis-specific protein interacts functionally with eukaryotic translation elongation factor eEF1A, a putative oncoprotein.. Int J Cancer.

[7] Cao H, Zhu Q, Huang J, Li B, Zhang S (2009). Regulation and functional role of eEF1A2 in pancreatic carcinoma.. Biochem Biophys Res Commun.

[8] Knudsen SM, Frydenberg J, Clark BF, Leffers H (1993). Tissue-dependent variation in the expression of elongation factor-1 alpha isoforms: isolation and characterisation of a cDNA encoding a novel variant of human elongation-factor 1 alpha.. Eur J Biochem.

[9] Lee S, Francoeur AM, Liu S, Wang E (1992). Tissue-specific expression in mammalian brain, heart, and muscle of S1, a member of the elongation factor-1 alpha gene family.. J Biol Chem.

[10] Chambers DM, Peters J, Abbott CM (1998). The lethal mutation of the mouse wasted (wst) is a deletion that abolishes expression of a tissue-specific isoform of translation elongation factor 1alpha, encoded by the Eef1a2 gene.. Proc Natl Acad Sci U S A.

[11] Browne GJ, Proud CG (2002). Regulation of peptide-chain elongation in mammalian cells.. Eur J Biochem.

[12] Amiri A, Noei F, Jeganathan S, Kulkarni G, Pinke DE (2007). eEF1A2 activates Akt and stimulates Akt-dependent actin remodeling, invasion and migration.. Oncogene.

[13] Ejiri S (2002). Moonlighting functions of polypeptide elongation factor 1: from actin bundling to zinc finger protein R1-associated nuclear localization.. Biosci Biotechnol Biochem.

[14] Ruest LB, Marcotte R, Wang E (2002). Peptide elongation factor eEF1A-2/S1 expression in cultured differentiated myotubes and its protective effect against caspase-3-mediated apoptosis.. J Biol Chem.

[15] Condeelis J (1995). Elongation factor 1 alpha, translation and the cytoskeleton.. Trends Biochem Sci.

[16] Bektas M, Nurten R, Gurel Z, Sayers Z, Bermek E (1994). Interactions of eukaryotic elongation factor 2 with actin: a possible link between protein synthetic machinery and cytoskeleton.. FEBS Lett.

[17] Gross SR, Kinzy TG (2005). Translation elongation factor 1A is essential for regulation of the actin cytoskeleton and cell morphology.. Nat Struct Mol Biol.

[18] Chuang SM, Chen L, Lambertson D, Anand M, Kinzy TG (2005). Proteasome-mediated degradation of cotranslationally damaged proteins involves translation elongation factor 1A.. Mol Cell Biol.

[19] Shamovsky I, Ivannikov M, Kandel ES, Gershon D, Nudler E (2006). RNA-mediated response to heat shock in mammalian cells.. Nature.

[20] Anand N, Murthy S, Amann G, Wernick M, Porter LA (2002). Protein elongation factor EEF1A2 is a putative oncogene in ovarian cancer.. Nat Genet.

[21] Chang R, Wang E (2007). Mouse translation elongation factor eEF1A-2 interacts with Prdx-I to protect cells against apoptotic death induced by oxidative stress.. J Cell Biochem.

[22] Qi CF, Zhou JX, Lee CH, Naghashfar Z, Xiang S (2007). Anaplastic, plasmablastic, and plasmacytic plasmacytomas of mice: relationships to human plasma cell neoplasms and late-stage differentiation of normal B cells.. Cancer Res.

[23] Zhang JQ, Okumura C, McCarty T, Shin MS, Mukhopadhyay P (2004). Evidence for selective transformation of autoreactive immature plasma cells in mice deficient in Fasl.. J Exp Med.

[24] Lee CH, Melchers M, Wang H, Torrey TA, Slota R (2006). Regulation of the germinal center gene program by interferon (IFN) regulatory factor 8/IFN consensus sequence-binding protein.. J Exp Med.

[25] Shin DM, Shaffer DJ, Wang H, Roopenian DC, Morse HC (2008). NOTCH is part of the transcriptional network regulating cell growth and survival in mouse plasmacytomas.. Cancer Res.

[26] Morse HC, McCarty T, Qi CF, Torrey TA, Naghashfar Z (2003). B lymphoid neoplasms of mice: characteristics of naturally occurring and engineered diseases and relationships to human disorders.. Adv Immunol.

[27] Qi CF, Xiang S, Shin MS, Hao X, Lee CH (2006). Expression of the cyclin-dependent kinase inhibitor p27 and its deregulation in mouse B cell lymphomas.. Leuk Res.

[28] Akilesh S, Shaffer DJ, Roopenian D (2003). Customized molecular phenotyping by quantitative gene expression and pattern recognition analysis.. Genome Res.

[29] Park ES, Shaughnessy JD, Gupta S, Wang H, Lee JS (2007). Gene expression profiling reveals different pathways related to Abl and other genes that cooperate with c-Myc in a model of plasma cell neoplasia.. BMC Genomics.

[30] Keats JJ, Fonseca R, Chesi M, Schop R, Baker A (2007). Promiscuous mutations activate the noncanonical NF-kappaB pathway in multiple myeloma.. Cancer Cell.

[31] Jeganathan S, Morrow A, Amiri A, Lee JM (2008). Eukaryotic elongation factor 1A2 cooperates with phosphatidylinositol-4 kinase III beta to stimulate production of filopodia through increased phosphatidylinositol-4,5 bisphosphate generation.. Mol Cell Biol.

[32] Jeganathan S, Lee JM (2007). Binding of elongation factor eEF1A2 to phosphatidylinositol 4-kinase beta stimulates lipid kinase activity and phosphatidylinositol 4-phosphate generation.. J Biol Chem.

[33] Panasyuk G, Nemazanyy I, Filonenko V, Negrutskii B, El'skaya AV (2008). A2 isoform of mammalian translation factor eEF1A displays increased tyrosine phosphorylation and ability to interact with different signalling molecules.. Int J Biochem Cell Biol.

[34] Kim MJ, Si F, Kim SJ, Hong SB, Hwang JI (1999). The SH2-SH2-SH3 domain of phospholipase C-gamma1 directly binds to translational elongation factor-1alpha.. Mol Cells.

[35] Lee S, LeBlanc A, Duttaroy A, Wang E (1995). Terminal differentiation-dependent alteration in the expression of translation elongation factor-1 alpha and its sister gene, S1, in neurons.. Exp Cell Res.

[36] Tomlinson VA, Newbery HJ, Bergmann JH, Boyd J, Scott D (2007). Expression of eEF1A2 is associated with clear cell histology in ovarian carcinomas: overexpression of the gene is not dependent on modifications at the EEF1A2 locus.. Br J Cancer.

[37] Qi CF, Shin DM, Li Z, Wang H, Feng J (2010). Anaplastic plasmacytomas: relationships to normal memory B cells and plasma cell neoplasms of immunodeficient nd autoimmune mice.. J Pathol.

[38] Landgren O, Kyle RA, Pfeiffer RM, Katzmann JA, Caporaso NE (2009). Monoclonal gammopathy of undetermined significance (MGUS) consistently precedes multiple myeloma: a prospective study.. Blood.

[39] Yamashita S, Tsujino Y, Moriguchi K, Tatematsu M, Ushijima T (2006). Chemical genomic screening for methylation-silenced genes in gastric cancer cell lines using 5-aza-2′-deoxycytidine treatment and oligonucleotide microarray.. Cancer Sci.

[40] Aaronson DS, Horvath CM (2002). A road map for those who don't know JAK-STAT.. Science.

[41] Catlett-Falcone R, Landowski TH, Oshiro MM, Turkson J, Levitzki A (1999). Constitutive activation of Stat3 signaling confers resistance to apoptosis in human U266 myeloma cells.. Immunity.

[42] Chiarle R, Simmons WJ, Cai H, Dhall G, Zamo A (2005). Stat3 is required for ALK-mediated lymphomagenesis and provides a possible therapeutic target.. Nat Med.

[43] Ferrajoli A, Faderl S, Ravandi F, Estrov Z (2006). The JAK-STAT pathway: a therapeutic target in hematological malignancies.. Curr Cancer Drug Targets.

[44] Neubauer H, Cumano A, Muller M, Wu H, Huffstadt U (1998). Jak2 deficiency defines an essential developmental checkpoint in definitive hematopoiesis.. Cell.

[45] Quintanilla-Martinez L, Kremer M, Specht K, Calzada-Wack J, Nathrath M (2003). Analysis of signal transducer and activator of transcription 3 (Stat 3) pathway in multiple myeloma: Stat 3 activation and cyclin D1 dysregulation are mutually exclusive events.. Am J Pathol.

[46] Kovalchuk AL, Qi CF, Torrey TA, Taddesse-Heath L, Feigenbaum L (2000). Burkitt lymphoma in the mouse.. J Exp Med.

[47] Morse HC, Qi CF, Chattopadhyay SK, Hori M, Taddesse-Heath L (2001). Combined histologic and molecular features reveal previously unappreciated subsets of lymphoma in AKXD recombinant inbred mice.. Leuk Res.

[48] Fredrickson TN, Lennert K, Chattopadhyay SK, Morse HC, Hartley JW (1999). Splenic marginal zone lymphomas of mice.. Am J Pathol.

[49] Potter M, Wiener F (1992). Plasmacytomagenesis in mice: model of neoplastic development dependent upon chromosomal translocations.. Carcinogenesis.

