# Supplementary material for: Eef1a2 Promotes Cell Growth, Inhibits Apoptosis and Activates JAK/STAT and AKT Signaling in Mouse Plasmacytomas
Source: PLoS One. 2010 May 21;5(5):e10755. doi: 10.1371/journal.pone.0010755 (PMC2873962; doi:10.1371/journal.pone.0010755)
Supplement: Figure S1 — Specific knockdown Eef1a2 expression in PCT cell lines. (A) qPCR analyses of Eef1a1 and Eef1a2 expression levels in transiently transfected PCT-AP cell line with four plasmids specifically targeting Eef1a2 (shRNA-1,2,3,4) and a control plasmid (shRNA-C). (B) Eef1a1 and Eef1a2 transcripts (left) and eEF1A2 protein (right) levels were analyzed by qPCR and western blotting, respectively, in stably transfected ABPC4 cells with a plasmid expressing shRNA-3 specifically targeting Eef1a2 and a plasmid expressing control shRNA-C. Error bar = ± S.E. **p<0.01. (0.02 MB PDF) [file pone.0010755.s001.pdf]

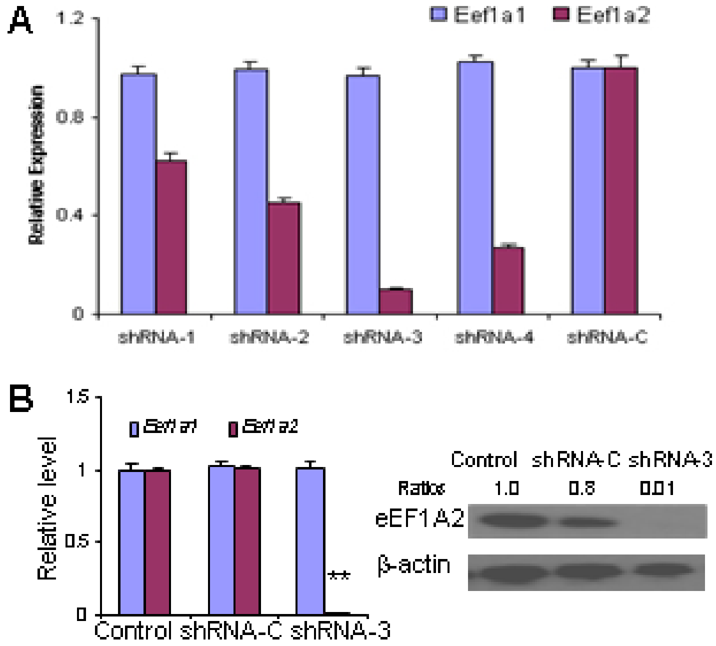

Supplemental Figure 1. Specific knockdown *Eef1a2* expression in PCT cell lines. (A) qPCR analyses of *Eef1a1* and *Eef1a2* expression levels in transiently transfected PCT-AP cell line with four plasmids specifically targeting *Eef1a2* (shRNA-1,2,3,4) and a control plasmid (shRNA-C). **(B)** *Eef1a1 and Eef1a2* transcripts (left) and eEF1A2 protein (right) levels were analyzed by qPCR and western blotting, respectively, in stably transfected ABPC4 cells with a plasmid expressing shRNA-3 specifically targeting *Eef1a2* and a plasmid expressing control shRNA-C. Error bar = ± S.E. **p < 0.01
