# Supplementary material for: Eef1a2 Promotes Cell Growth, Inhibits Apoptosis and Activates JAK/STAT and AKT Signaling in Mouse Plasmacytomas
Source: PLoS One. 2010 May 21;5(5):e10755. doi: 10.1371/journal.pone.0010755 (PMC2873962; doi:10.1371/journal.pone.0010755)
Supplement: Figure S2 — (A) The frequency of EdU-postive cells in the Eef1a2 shRNA-3 expressing, control shRNA-C expressing cells and control cells were analyzed by flow cytometry. Error bar = ± S.E. **p<0.01. (B) Apoptotic cells were analyzed by flow cytometry in cells transiently transfected with Eef1a2 shRNAs and shRNA-C after culturing in serum-free medium for 48 hours. (0.05 MB PDF) [file pone.0010755.s002.pdf]

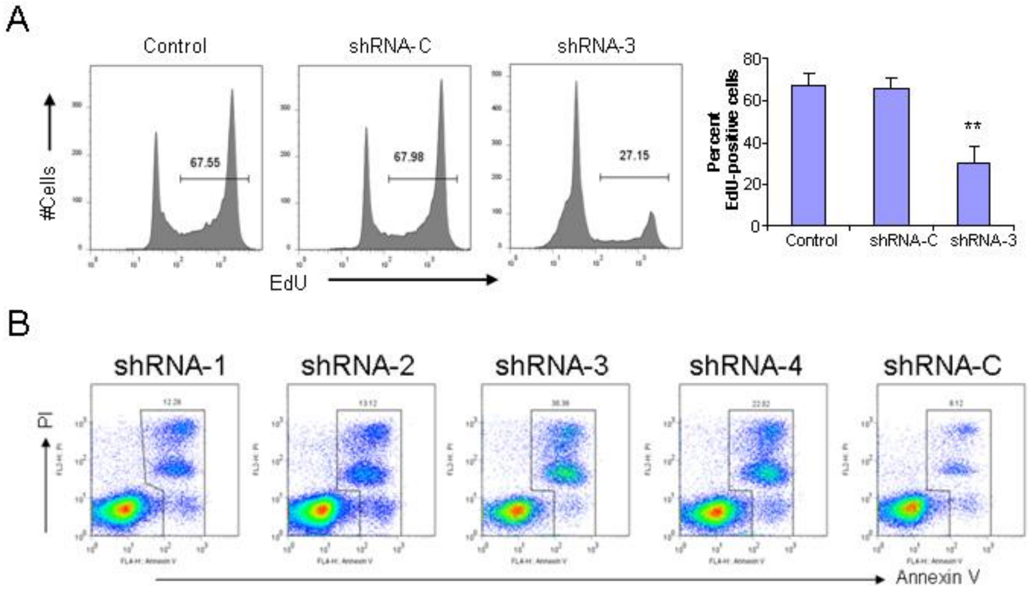

Supplemental Figure 2. (A) The frequency of EdU-postive ABPC4 cells in the *Eef1a2* shRNA-3 expressing, control shRNA-C expressing cells and control cells were analyzed by flow cytometry. Error bar = ± S.E. **p < 0.01 **(B)** Apoptotic cells were analyzed by flow cytometry in PCT-AP cell line transiently transfected with *Eef1a2* shRNAs and shRNA-C after culturing in serum-free medium for 48 hours.
