# Supplementary material for: Eef1a2 Promotes Cell Growth, Inhibits Apoptosis and Activates JAK/STAT and AKT Signaling in Mouse Plasmacytomas
Source: PLoS One. 2010 May 21;5(5):e10755. doi: 10.1371/journal.pone.0010755 (PMC2873962; doi:10.1371/journal.pone.0010755)
Supplement: Table S1 — Primers used for qPCR. (0.08 MB PDF) [file pone.0010755.s003.pdf]

Supplemental Table 1. Primers used for qPCR

| | Gene | Primer sequences |
|---|---|---|
| Human | *EEF1A1* | 5’-AAATTGGCTACAACCCCGACA-3’ |
| | | 5’-TCCCTTGAACCAAGGCATGTTA-3’ |
| | *EEF1A2* | 5’-CCCTCACA CTCCCAGCAAAAT-3’ |
| | | 5’- TTTGTAGATGAGGTGGCCCGT-3’ |
| | *β-actin* | 5’-CGTGGACATCCGCAAAGAC-3’ |
| | | 5’-TGCATCCTGTCGGCAAT-3’ |
| | *Eef1a1* | 5’-GTCAGAACGCAGGTGTTGTGAA-3’, |
| | | 5’-CCGGAATCTA CGTGTCCGATTA-3’; |
| | *Eef1a2* | 5’-TACCCTCAACCCCAAACCAGA-3’, |
| | | 5’-GGCCAATGACCACAATGTTGAT-3’. |
| | *Tgfbr2* | 5’-CGTCCCGCTGCAATGC-3’ |
| | | 5’-CGCACCTTGGAACCAAATG-3’ |
| | *Bcl2* | 5’-AAGGGCTTCACACCCAAATCT-3’ |
| | | 5’-TTCTACGTCTGCTTGGCTTTGA-3’ |
