# Supplementary material for: Eef1a2 Promotes Cell Growth, Inhibits Apoptosis and Activates JAK/STAT and AKT Signaling in Mouse Plasmacytomas
Source: PLoS One. 2010 May 21;5(5):e10755. doi: 10.1371/journal.pone.0010755 (PMC2873962; doi:10.1371/journal.pone.0010755)
Supplement: Table S2 — Signaling classification for differentially expressed genes by Eef1a2 knock-down. (0.10 MB PDF) [file pone.0010755.s004.pdf]

Supplemental Table 2. Signaling classification

for differentially expressed genes by

Signaling	Odds	*p*
Notch	1.35	0.59
Wnt	1.01	1
G-protein	2.33	0.20
Shh	1.14	1
JAK-STAT*	4.16	0.01
NF-kB	1.28	0.48

The significant genes that distinguish *Eef1a2* knockdown cells from those of control cells were identified by *t*-test at $p < 0.05$ and then classified into functional categories by GO. Enrichment of differentially expressed genes in each category was examined by Fisher's exact test.

Supplemental Table 1. Primers used for qPCR

	Gene	Primer sequences
Human	*EEF1A1*	5'-AAATTGGCTACAACCCCGACA-3'
		5'-TCCCTTGAACCAAGGCATGTTA-3'
	EEF1A2	5'-CCCTCACA CTCCCAGCAAAAT-3'
		5'- TTTGTAGATGAGGTGGCCCGT-3'
	β-actin	5'-CGTGGACATCCGCAAAGAC-3'
		5'-TGCATCCTGTCGGCAAT-3'
	Eef1a1	5'-GTCAGAACGCAGGTGTTGTGAA-3',
		5'-CCGGAATCTA CGTGTCCGATTA-3';
	Eef1a2	5'-TACCCTCAACCCCAAACCAGA-3',
		5'-GGCCAATGACCACAATGTTGAT-3'.
	Tgfbr2	5'-CGTCCCGCTGCAATGC-3'
		5'-CGCACCTTGGAACCAAATG-3'
	Bcl2	5'-AAGGGCTTCACACCCAAATCT-3'
		5'-TTCTACGTCTGCTTGGCTTTGA-3'
